# Double dehiscence (Superior semicircular canal and tegmen tympani) in the epicampaniform period (Arboli type)

**DOI:** 10.1007/s00405-025-09445-2

**Published:** 2025-05-20

**Authors:** AI. Cisneros-Gimeno, A. García-Barrios, S. Baena-Pinilla, J. Obón-Nogués, R. Gómez-Miranda, J. Whyte-Orozco, M. Botella-López

**Affiliations:** 1 Department of Human Anatomy and Histology, Faculty of Medicine, University of Zaragoza, C/ Domingo Miral, s/n, Zaragoza, 50009 Spain; 2 Antecessor B51_23D (Government of Aragon), Barcelona, Spain; 3 Medical and Genetic Research Group (GIIS099), Aragon Health Research Institute, Zaragoza, Spain; 4 Centro de Radiodiagnóstico Gómez Pereda, Zaragoza, Spain; 5 Department of Legal Medicine, Toxicology and Physical Anthropology, School of Medicine, University of Granada, Granada, Spain

**Keywords:** Superior semicircular canal dehiscence, Tegmen tympani dehiscence, Temporal bone pneumatization, Iberian peninsula, 1800–1700 BC

## Abstract

**Purpose:**

Although the superior semicircular canal dehiscence syndrome was described at the end of the 20th century, we want to check if it is a pathology that has existed since ancient times, through the anthropological study of bone remains.

**Methods:**

We have carried out an anthropological and radiological study (CT scan) of 8 skulls found in caves, as secondary burials of the Arbolí type epicampaniform culture (1800 − 1700 BC) on the Iberian Peninsula.

**Results:**

The 8 skulls (16 temporal bones) show a grade 4 degree of pneumatisation or hyperpneumatization. One of these skulls, belonging to a male subject of around 25–30 years of age, shows a double dehiscence (superior semicircular canal and tegmen tympani) on the right side, and a possible congenital muscular torticollis on the same side.

**Conclusion:**

Superior semicircular canal dehiscence syndrome already existed in an inhabitant from 1800 − 1700 BC (Iberian Peninsula). This is the first case in which the association of both dehiscences (superior semicircular canal and tegmen tympani) has been demonstrated.

## Introduction

In 1969 an archaeologist from Borja (Zaragoza-Spain), Gregorio Viamonte, found some caves; 3 years later, the first skulls were found in these caves, and these skulls were those of children. These findings were part of a group of secondary burials, and with some exceptions, the only findings were skulls from people of different ages and sexes. These skulls were generally found on the floor of the cave or in holes in the walls of different caves located in “Muela de Borja” (Spain), and the skulls were from individuals from the epicampaniform culture of the Arbolí type (1800 BC–1700 BC) on the Iberian Peninsula [1].

Superior semicircular canal dehiscence (SSCD) is an inner ear disorder that was first described by Minor in 1998, and it was the last otological syndrome that was described in the 20th century [2]. The radiological assessment of SSCD is based on the absence of the bony layer that separates the intracranial space and superior semicircular canal (SSC) in reconstructions of the Pösch plane (the plane of the SSC of each ear). Therefore, the canal lumen is open to the middle cranial fossa or the superior petrosal sinus. This syndrome is quite unknown in palaeopathology, and only one case has been described in an Egyptian mummy [3].

This manuscript describes the main paleopathological findings of one of the 8 skulls studied from “Muela de Borja”, which were from people of different ages and sexes, with a primary focus on its particular pneumatization and on the unilateral dehiscence in the superior semicircular canal, which was associated with a tegmen tympani dehiscence on the same side. This is the first case described from an ancient population.

## Materials and methods

A morphological study was carried out on 8 skulls (16 temporal bones) from the epicampaniform period (Arboli type) from Bell Beaker inhabitants of the “Muela de Borja”. The skulls are preserved in the Museum of Zaragoza (Govern of Aragon). We subsequently focused mainly on the study of one of these skulls.

Radiological studies with CT scans were performed at the Centro de Radiodiagnóstico Gómez Pereda (Zaragoza) with General Electric ^®^ Optima CT520 series equipment. Helical acquisition was performed by obtaining images in the coronal plane after positioning the skull; these images represented 0.625-mm-thick slices, and sagittal reconstruction was obtained with a bone window of 2-mm-thick slices. The reconstructions were generated in the Pöschl plane.

To classify the pneumatization of the temporal bone, we used the classification system described by Allam (1969), which divides the temporal bone into the mastoid, perilabyrinthine, petrosal apex, and accessory regions (zygomatic, squamosal, occipital, and styloid) [4].

## Results

After reviewing the CT scans of 16 temporal bones that remained intact in the 8 skulls, several findings should be highlighted. In all of the skulls, there was great pneumatization in the temporal bone, mastoid, petrous process, temporal scale, and labyrinth. We classified this pneumatization as grade 4 or hyperpneumatization. Other bones of the skulls, such as the occipital bone and clivus, did not appear normal. The skull from a 25-30-year-old male had a bone defect in the right unilateral superior semicircular canal. This superior semicircular canal showed dehiscence located at the apical level and progressive thinning of the bone until the dehiscence was observed, leaving a communication zone between the middle cranial fossa and the lumen of the canal. A large degree of supra-, trans- and infralabyrinthine pneumatization was also observed around the canal, connecting the labyrinth with the tympanic cavity and the petrosal apex, and there were bony islands that corresponded to translabyrinthine cells. The size of this area of dehiscence was 0.12 cm. Serial CT slices of the dehiscent canal were used to observe that the anterior and posterior parts of the canal were papyraceous (0.1 cm). The semicircular canal on the other side was intact, with a bony island, as were the posterior semicircular canals, which were pneumatized.

**Fig. 1:**
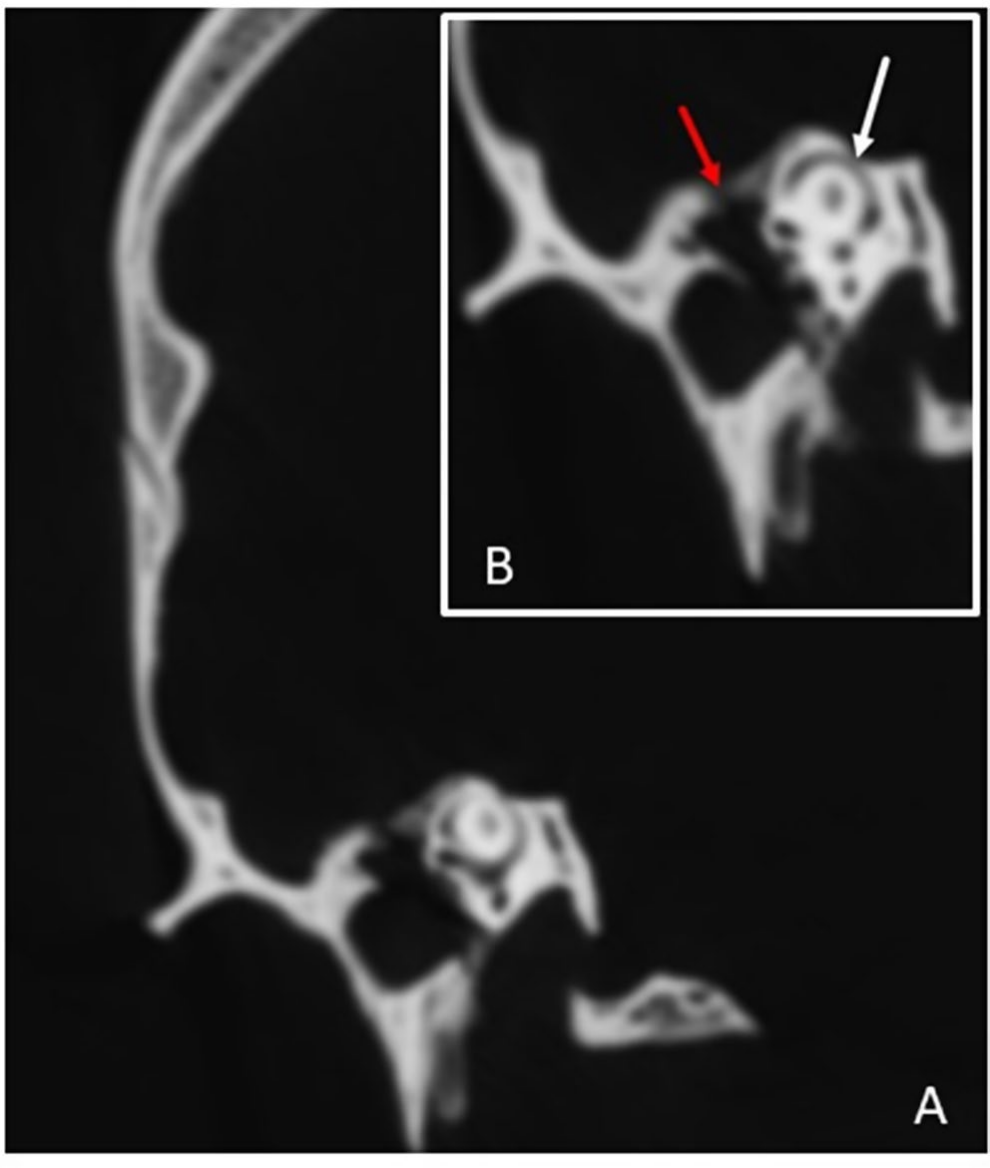
(**a**,**b**) Dehiscences in both structures, superior semicircular canal (white arrow) and tegmen tympani (red arrow)

After examining the whole petrous and searching for other possible associated dehiscence between the structures that had the same embryological origin, such as the otic capsule, we detected dehiscence (0.26 cm) of the tegmen tympani on the same side that communicated with the tympanic cavity and endocranium, indicating a unifocal defect (grade 4) (Fig. 1a, b). The rest of the structures, including the other semicircular canals (posterior and lateral), internal auditory canal, glenoid fossa, geniculate ganglion, jugular bulb, carotid duct, and the eustachian tube, did not show any type of bone defect.

**Fig. 2:**
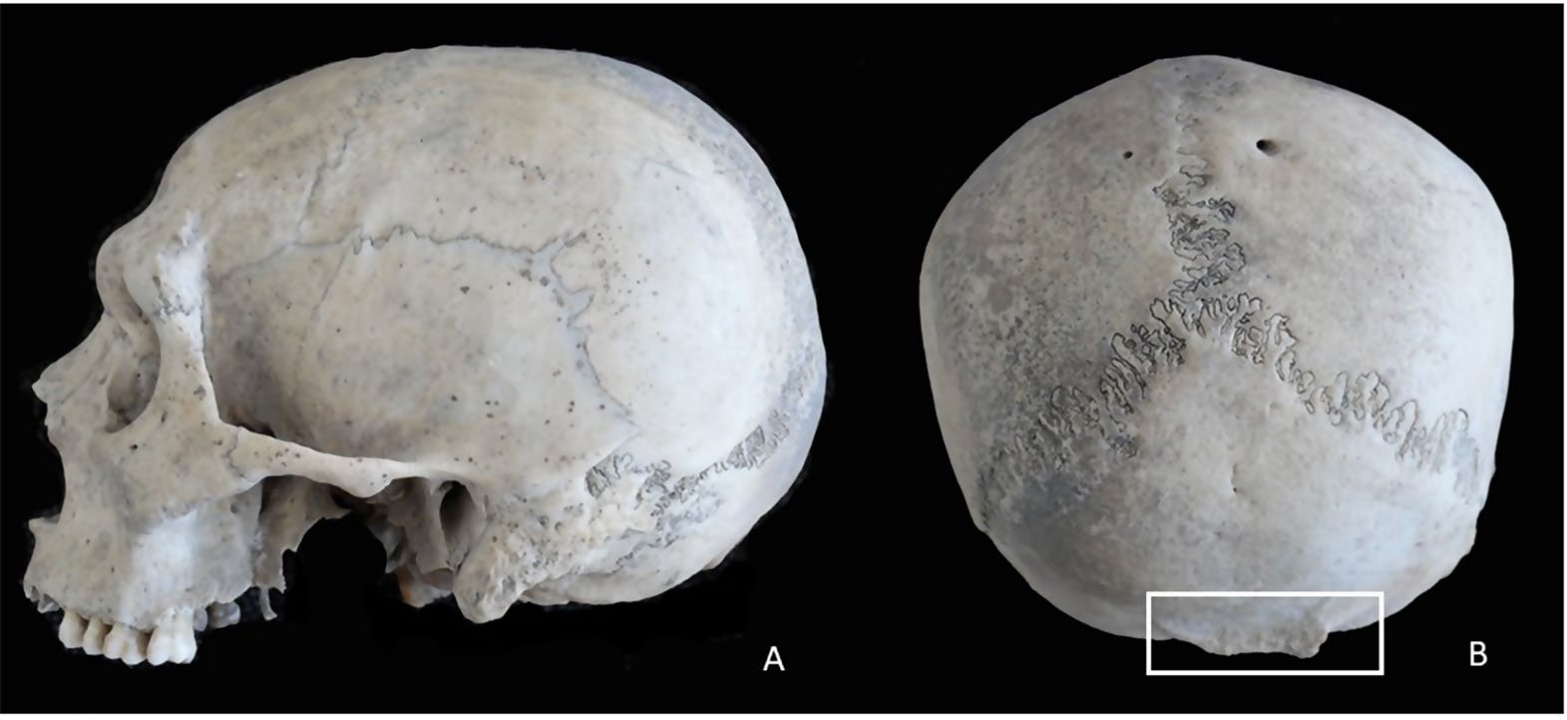
(**a**,**b**) Skull with hypertrophied inion and lateralized to the right (left lateral and posterior vision)

We investigated the presence of other possible cranial alterations in this subject even though their embryological origin was different from that of the two dehiscent structures. We located the hypertrophied inion, which was very lateralized to the right, and suggest it was compatible with a possible congenital muscular torticollis (Fig. 2a, b).

## Discussion

The unilateral superior semicircular canal dehiscence described on the right side of this male subject was not the first case to be found in historical skulls, as one was observed in an ancient Egyptian mummy who was a male subject between 45 and 50 years old and dated between 2000 and 2400 BC [3]. Considering the images, we obtained in our study and the accompanying text, a question arises regarding whether there was true dehiscence or just subdehiscence, as it was a very thin bone layer corresponding to a papyraceous pattern [5].

We can affirm that this skull is the first from ancient populations of the epicampaniform period (arboreal type) that inhabited Europe to be described. In addition to an association with tegmen tympani dehiscence, this is the oldest case of double dehiscence described in palaeopathology.

The existence of dehiscence of the superior semicircular canal and the tegmen tympani together, despite being a well-established clinical entity, was published only recently. In fact, the first description of its presence was made by Minor in 2000 [6]. Authors such as Brantberg [7], Pletcher [8], Friedland [9], Mahendran [10], Martin [11], and Suryanayanan [12] described the presence of dehiscence in isolated patients, whereas others such as Crovetto [13], El Haidi [14], and Whyte [15] reported the presence of this type of double dehiscence from CT images, with incidences varying between 36%, 56%, and 34.78%, respectively. In our study, the percentage was 12.5% (1/8), which is lower than other reported percentages. More population studies are needed to verify the true incidence in subjects who have lived in different periods and to compare them with the current incidence.

We believe that the explanation for double dehiscence given by Fraile in 2016 [16], when he demonstrated the same embryological origin for both entities, is that the periosteum that covers the roof of the superior semicircular canal continues with that of the tegmen tympani. In addition, the apical canalicular nucleus of the superior semicircular canal intervenes by extension and growth in the endochondral ossification of the tegmental process. The embryological origin justifies the coexistence of both dehiscences on the same side of the subject. Furthermore, to reaffirm the theory of the congenital origin of these dehiscences, the fact that the skull also presents alterations in the incision is compatible with congenital muscular torticollis.

Another noteworthy finding was the presence of a bony island of the superior semicircular canal, which we believe corresponded to translabyrinthine cells that had not yet ossified and passed through the subarcuate fossa.

This type of bone defect, such as dehiscence, is overlooked in anthropological studies for two main reasons. The first is poor preservation of the bone, which makes it difficult to study dehiscences, and second, as in this case, dehiscence is a very recently diagnosed otorhinolaryngological syndrome, studies in ancient populations are lacking because anthropologists are unaware of its existence or do not know how to diagnose this type of alteration.

The little information that is available from anthropological studies of Egyptian mummies should be reviewed because the reduced thickness of the slices with new CT scanning systems allows the differentiation between true dehiscence and subdehiscence (papillary pattern).

The detailed study of the skull of this subject has provided us with important information about the social characteristics of the group in which he lived, since despite the malformations he had and his possible symptoms, he reached adulthood. This indicates that he was very well cared for, and we can even think that he could have been someone of important social relevance or from a family of an important social class for the community; however, we cannot be sure of the latter, because these skulls were part of a secondary burial inside caves and were found without any grave goods.

If studies of certain bone alterations, defects, or malformations are not carried out on ancient skulls, there could be loss of data related to the societies in which the subjects lived and their coexistence with other individuals.

## Conclusion

Superior semicircular canal dehiscence syndrome, although one of the last syndromes to be described during the 20th century, already existed in an inhabitant of the epicampaniform period (Arboli type) on the Iberian Peninsula from 1800 to 1700 BC. This is the first case in which the association of both dehiscences (superior semicircular canal and tegmen tympani) has been demonstrated.
